# Life in a Chinese Hospital

**Published:** 1920-09-25

**Authors:** 


### LIFE IN A CHINESE HOSPITAL.

BY A NURSE ON THE SPOT.

THOSE nurses who have never left England will find it hard to imagine what hospital life in a foreign country is like. Our little hospital in Tai-yuan-fu (Shansi) is always full of Chinese women and children—full to overflowing—because although we have only thirty-seven beds we often have in as many as fifty patients. To have a hospital without several doctors, I fancy, is unheard of in England, but there just now we have no European doctor, and so we two English nurses have to do many things that we would not do if we had a doctor at the other end of the telephone. We have two foreign-trained Chinese doctors, but they, like us, long for another medical man.

Patients come from many days' journey away, from the near villages, and from the city where we live and do our work. They are ignorant and very few of them can read; in fact, we hardly ever get a woman who can read. For their "illnesses"—the majority of them are needing surgical treatment—they themselves have medicine which they give in large doses warranted to kill or cure, but to do the work quickly. We also have a number of midwifery cases. In 1919 we had eighty children born in hospital; of those the larger number were abnormalities and included twelve Cæsarean sections.

These poor women suffer fearfully, many of them coming to us after being three or four days in labour, and perhaps coming a long journey in a litter carried by two mules, and some even travel over very rough roads in a springless cart. The worst case I remember was a woman who came, after being eleven days in labour, with the report that the native midwife said it was not a baby but a bony and hairy substance. Needless to say, this midwife had done terrible harm to both mother and child. How she managed it we could not make out, but she evidently had used some sharp instrument and had succeeded in removing some of the child's skull bones. The child was delivered with forceps, and the native midwife, who saw the child at the hospital, was told of the wrong she had done and advised to bring any other such case to us as early as possible. These women are absolutely ignorant of ordinary cleanliness, to say nothing of surgical cleanliness. They know nothing of anything relating to child-birth, and some of them look and are villains.

Every Chinese woman rejoices at the birth of a son, but needs to be condoled with when a daughter is born. It caused some consternation a short time ago to hear one husband say to his wife, "If it's a son I shall buy you a fur coat, but if it's a daughter I shall get another wife." Another patient wept because her husband on seeing her for the first time after their little girl was born asked her in a disinterested way what she (his wife) intended doing with the child, as he did not want a baby girl.

We get numbers of women who come in to break off the opium habit: some of them have taken it for many years, usually because they had some pain or disease that their native "doctor" could not cure because he did not understand it, and by constantly taking opium, and in many cases taking it in large doses up to as much as an ounce per day, it is easy to understand the harm that they inflict upon themselves. We had a patient in hospital in 1918 who had eaten opium for twenty years; she brought with her a baby that looked like a famine child—pale, thin, and the acme of misery. We asked her what she had done to him and she said: "Well, from his birth he cried a lot, and to keep him quiet I puffed opium smoke down his throat." The mother and baby stayed until they were quite well and both went home looking different people.

On one occasion we were having dinner and heard somebody shouting in the courtyard. We were told that one of the opium patients had died, and they were going to get her husband. On arriving in the ward I found several of the hospital servants standing round the bed, all saying she had died. The patient, true enough, was pretending to be dead, but her pulse was beating as nicely as my own. You should have seen how surprised the watchers were when I just touched her eye with my finger and she nearly jumped off the bed.

We succeeded in getting our first Chinese nurse in 1914. From a health point of view I do not suppose any doctor in England would have passed her, as we first knew her through coming to us to have glands removed from her neck. This girl was well educated and her father was a Professor in the Shansi University. The second girl we got would also have failed to pass the doctor in England, as she was very undersized, but she, too, was well educated and her brother held an official position. Like many another woman, she objected to growing old, and after deciding that to be nineteen years of age was very nice she made up her mind to remain so for many years. Although we know she is about thirty, she is still nineteen. These are the only nurses we have had that really come up to the educational standard required.

Three of our nurses have had their certificates, and we made a great day of the ceremony. These nurses, in addition to their ordinary training, have taken midwifery, and a nurse who was on a visit to Tai Yuan examined them in this subject and gave it as her opinion that they came up to the standard of our English C.M.B.. The standard we have reached now was not reached in a few weeks or even a few months. I remember the time when to get our Chinese nurses to comb a patient's hair we had first to do it ourselves; it was the same with bathing them: they looked upon it as doing the work of a servant at least, or even a slave.

But little by little the nurses we have are beginning to see how much quicker is the improvement of patients who are well nursed, and we are looking forward to the time when we shall have more nurses and are able ourselves to spend more time in training them.

